# Choosing not to act: Neural bases of the development of intentional inhibition

**DOI:** 10.1016/j.dcn.2014.08.006

**Published:** 2014-08-20

**Authors:** Margot A. Schel, K. Richard Ridderinkhof, Eveline A. Crone

**Affiliations:** a Institute of Psychology, Leiden University, Leiden, the Netherlands; b Leiden Institute for Brain and Cognition (LIBC), Leiden, the Netherlands; c Department of Psychology, University of Amsterdam, Amsterdam, the Netherlands; d Amsterdam Brain & Cognition (ABC), University of Amsterdam, Amsterdam, the Netherlands

**Keywords:** Development, Response inhibition, Self-control, Volition, fMRI, Fronto-basal ganglia network

## Abstract

Choosing not to act, or the ability to intentionally inhibit your actions lies at the core of self-control. Even though most research has focused on externally primed inhibition, an important question concerns how intentional inhibition develops. Therefore, in the present study children (aged 10–12) and adults (aged 18–26) performed the marble task, in which they had to choose between acting on and inhibiting a prepotent response, while fMRI data were collected. Intentional inhibition was associated with activation of the fronto-basal ganglia network. Activation in the subthalamic nucleus and dorsal fronto-median cortex, regions which have previously been associated with intentional inhibition, did not differ between intentional inhibition and intentional action. Even though both children and adults intentionally inhibited their actions to a similar extent, children showed more activation in the fronto-basal ganglia network during intentional inhibition, but not in the subthalamic nucleus and dorsal fronto-median cortex. Furthermore, a positive relation between self-reported impulsivity and intentional inhibition was observed. These findings have important implications for our understanding of disorders of impulsivity, such as ADHD, which are associated with poor self-control abilities.

## Highlights

• Intentional inhibition shows an early development.
• Intentional inhibition is associated with fronto-basal ganglia network activation.
• Fronto-basal ganglia activity is stronger in children than adults.
• Self-reported impulsivity and intentional inhibition are positively correlated.

## 1 Introduction

Self-control abilities are of critical importance for successful functioning across the life span. A classical experiment to test self-control is the marshmallow experiment, a delay of gratification test for preschool children in which children can choose between one marshmallow now or two marshmallows later (Mischel et al., 1989). Studies with the marshmallow experiment have shown that during the preschool ages there are large individual differences in the ability to control the immediate impulse to eat the first marshmallow and wait for the second marshmallow (Mischel et al., 1989) and experimental variations of this task have shown a developmental increase in controlling immediate impulses between childhood and adulthood (Christakou et al., 2011, Lee et al., 2013, Scheres et al., 2006). These changes are accompanied by neurodevelopmental changes. Recently, it has been shown that individuals who were less able to delay gratification when they were preschoolers, showed poorer self-control and reduced recruitment of the fronto-basal ganglia network during a response inhibition task in adulthood (Casey et al., 2011).

An important distinction which has been made in tasks that involve self-control, is whether the inhibitory process is externally or internally driven. Externally driven response inhibition involves the ability to interrupt an action when signaled by a cue in the environment, for example, a traffic light which turns red. Neuroscientific studies have shown that the fronto-basal ganglia network, with main nodes in right inferior frontal gyrus (rIFG), striatum, globus pallidus, and subthalamic nucleus (STN), is consistently involved during externally driven response inhibition (e.g. Aron et al., 2007, Aron and Poldrack, 2006, Forstmann et al., 2012, Jahfari et al., 2011, King et al., 2012). Developmental studies have also found evidence for the involvement of the fronto-basal ganglia network in externally driven response inhibition in children (Cohen et al., 2010, Ordaz et al., 2013). So far, most developmental neuroimaging studies have focused on the frontal component of the fronto-basal ganglia network, namely the rIFG (Luna et al., 2010). These studies have reported both increases (e.g. Ordaz et al., 2013, Rubia et al., 2007) and decreases (e.g. Booth et al., 2003, Durston et al., 2002) in inhibition related rIFG activation with age, but an age-related increase in rIFG activation and a decrease in activation in task-unspecific frontal regions appears to be the most common pattern (Crone and Dahl, 2012, Durston et al., 2006, Luna et al., 2010). Overall, these studies show a stabilization of response inhibition performance and its underlying neural correlates during adolescence (Luna et al., 2010).

A second, but understudied component of self-control is intentional inhibition. In daily life often both external cues and internal processes play a role in the decision to inhibit, although sometimes external cues are more important (such as when stopping at a traffic light) and other times internal processes are more prominent (such as when inhibiting yourself from taking another piece of chocolate). However, in contrast to externally driven inhibition, intentional inhibition is not triggered by an external cue. Instead, intentional inhibition has been defined as a late internally driven veto process, a final opportunity to inhibit before action execution (Brass and Haggard, 2008, Filevich et al., 2012, Haggard, 2008). Since intentional inhibition is not preceded by any external stimulus, and does not result in any overt behavior, intentional inhibition has proven difficult to investigate. Yet, this type of inhibition is involved in many of our day to day activities such as inhibiting yourself from taking another piece of chocolate, inhibiting pressing the send button when being on the verge of sending an angry email, or inhibiting scratching itchy skin caused by eczema. Delay of gratification studies have reported developmental improvements in the ability to intentionally inhibit the impulse to choose an immediate reward (Christakou et al., 2011, Lee et al., 2013, Scheres et al., 2006), but these tasks involve many different processes besides intentional inhibition, such as reward sensitivity and reward discounting. A neuroscience perspective can provide important insights into the development of intentional inhibition, as this provides a covert measure of a process, which occurs without a specific stimulus and without any behavioral outcomes. An important question concerns whether intentional inhibition is guided by the same neural network as externally driven inhibition, or whether intentional inhibition is associated with different underlying neural circuitry, which may develop during child and adolescent development.

In the present study, we used the child-friendly marble paradigm to examine the neural correlates of intentional action and inhibition in children. In this paradigm participants are instructed to freely decide between responding and inhibiting responding to a rolling marble. Children between 10 and 12 years of age were included to allow for comparison of the results to the externally driven response inhibition literature (Booth et al., 2003, Bunge et al., 2002, Durston et al., 2002, Rubia et al., 2007). Based on these prior findings, we expect to observe activation in the main nodes of the fronto-basal ganglia network (i.e. rIFG, striatum, globus pallidus, and STN) during intentional inhibition, comparable to what we have observed in adults (Schel et al., 2014). If developmental differences in internally driven inhibition share mechanisms with externally driven response inhibition (Bunge et al., 2002, Durston et al., 2002, Luna et al., 2010), then we may expect inhibition-related activation in the rIFG to be stronger for adults compared to children.

Recently, several studies have suggested that intentional inhibition is associated with increased activation in the dorsal fronto-median cortex (dFMC) (Brass and Haggard, 2007, Kühn et al., 2009), a region which was previously found to be more active in early adolescents when inhibiting a selfish impulse in a sharing task (van den Bos et al., 2011). Therefore, we examined whether more activity in the dFMC during intentional inhibition was observed in children compared to adults based on prior studies showing that this region is more active in 10–12-year-olds (Blakemore, 2008, Gunther Moor et al., 2012, van den Bos et al., 2011).

In order to validate our experimental measure of intentional inhibition, participants also completed the Barratt Impulsiveness Scale (Patton et al., 1995) outside of the scanner to obtain an estimate of impulsivity and self-control in daily life. We expect daily life impulsivity to be predictive of intentional action control performance.

## 2 Methods

### 2.1 Participants

Nineteen healthy right-handed children between 10 and 12 years of age (10 females, *M* = 11.56, *SD* = .83) and twenty-four healthy right-handed adults between 18 and 26 years of age (13 females, *M* = 21.49, *SD* = 2.36) participated in the experiment. The results from the adults have previously been published in a larger report on response inhibition (Schel et al., 2014). A chi-square test revealed no significant differences in gender distributions between age groups (*p* = .92). All participants had normal or corrected-to-normal vision, and no neurological or psychiatric impairments according to self- or parent-report. Informed consent was obtained for all participants and the study was approved by the Internal Review Board at Leiden University Medical Center. In accordance with the guidelines of the Leiden University Medical Center, all anatomical scans were reviewed by a radiologist. No anomalous findings were reported.

To obtain an estimate of cognitive functioning, children and adults completed the subtests similarities and block design of the Wechsler Intelligence Scale for Children (WISC) (Wechsler, 1981b) and the Wechsler Adult Intelligence Scale (WAIS) (Wechsler, 1981a) respectively. Estimated IQ scores were within the normal range (children: *M*= 111.32, *SD* = 9.94, adults: *M* = 111.33, *SD* = 6.93) and age groups did not differ in estimated IQ scores, *F* (1,42) = .00, *p* = .99, *η*^2^ = 1.04^−6^.

### 2.2 Task

**Fig. 1:**
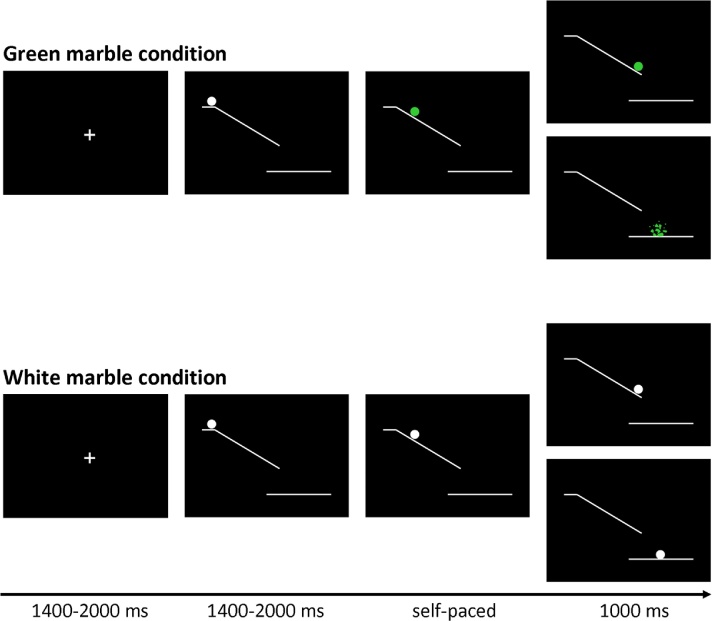
Trial structure of the marble task. Stimuli were presented at a black background. At the beginning of each trial a white marble on top of a ramp was presented. After a variable delay (jittered between 1400 and 2000 ms) the marble started to roll down the ramp, and could change color to green.

The marble task was adapted from Kühn et al. (2009). Each trial (see Fig. 1) started with the presentation of a fixation screen (white cross against a black background) with duration jittered between 1400 and 2000 ms. The fixation screen was followed by a screen showing a white ramp with a white marble on top presented against black background. After a variable duration of 1400–2000 ms the marble started rolling down the ramp and participants could stop the marble from crashing by pressing a button. Finally, a feedback screen, showing trial outcome, was presented for 1000 milliseconds. There were two task conditions: a green marble and a white marble condition.

In the green marble condition, the white marble changed to green as soon as it started rolling. The task was programmed in such a way that participants viewed 16 rapidly presented static pictures showing the marble at successive locations on the ramp, which was experienced as a rolling movement. Participants were instructed to stop the marble from crashing by pressing a response button with their right index finger. When participants were successful at stopping the marble, they were presented with a feedback screen showing the location where they had stopped the marble. When participants were not successful at stopping the marble, they were presented with a feedback screen showing a shattered marble beneath the ramp. The speed of the marble was adjusted by a staircase-tracking procedure. At the start of the experiment, the static pictures were presented for 30 milliseconds each. When participants were successful at stopping the marble the duration was decreased with 10 milliseconds, making the task more difficult. When participants were not successful at stopping the marble in time the duration was increased with 10 milliseconds, making the task easier. The staircase procedure was allowed to fluctuate between 20 and 80 milliseconds, allowing a response window between 320 and 1280 milliseconds.

In the white marble condition, the marble did not change color and participants were instructed to choose between responding and inhibiting. When participants responded, they were presented with a feedback screen showing the location where they had stopped the marble. When participants inhibited, they were shown a feedback screen showing the white marble at the bottom of the ramp. In order to motivate participants to balance the frequency of responding and inhibiting, they were told that the stopped and non-stopped marbles would fall in different baskets. Participants were instructed to collect an equal amount of marbles in each basket, but were not allowed to count or use a sequencing strategy; therefore, participants were instructed to make an independent decision every time the marble stayed white. At the end of each block participants were shown how many marbles they had collected in each basket. As will be described in the results section, the participants were successful in following the instruction to stop the marble on approximately 50% of the trials.

In order to give participants sufficient time to decide between responding and inhibiting the speed of the white marble rolling down the ramp was set considerably slower. The speed of the sequentially presented static white marble pictures was set to the speed currently reached in the green marble condition plus 30 milliseconds. Consequently the duration of the sequentially presented static white marble pictures was allowed to fluctuate between 50 and 110 milliseconds, allowing a response window between 800 and 1760 milliseconds.

The experiment consisted of three blocks of 80 trials, each block consisting of 48 green and 32 white marble trials. Trials were presented in a pseudo-randomized order so that each white marble trial was preceded by 0, 1, 2, or 3 green marble trials. The large proportion of green trials served two functions. First, the green trials lead to a prepotent tendency for action. Since the green marble trials were rather difficult, participants had to remain focused on the goal of responding quickly to the rolling marble, thus increasing the prepotency of responding. Also, due to the intermixed presentation of green and white trials participants could not predict when a white trial would be presented. Therefore participants had to be prepared to quickly respond to a green marble during the whole duration of the experiment, leaving the prepotency intact. This was desirable, so that intentional inhibition of action would involve a late brake on an already-prepared action, rather than a decision not to initiate action preparation. Second, the randomized interleaving of intentional (white) and instructed (green) trials also discouraged participants from strategically pre-deciding a pattern of intentional action, such as act-inhibit-act-inhibit, etc.

### 2.3 Barratt Impulsiveness Scale

Following the scanning session participants completed the Barratt Impulsiveness Scale (BIS-11) (Patton et al., 1995). The BIS-11 is a measure of impulsive traits consisting of three subscales: motor impulsivity (‘I act without thinking’), non-planning impulsivity (‘I’m not interested in the future, but in today’), and attentional impulsivity (‘I have difficulties sitting still during lectures’). For the child participants the questions were rephrased in an age-appropriate style (e.g. ‘I have difficulties sitting still in the classroom’ instead of ‘I have difficulties sitting still during lectures’).

### 2.4 Data acquisition

Scanning was performed with a standard whole-head coil on a 3.0 Tesla Philips scanner at the Leiden University Medical Center. The marble task consisted of 3 event-related runs, each lasting approximately 6 min. Functional data were acquired using T2*-weighted echo-planar imaging (EPI). The first 2 volumes of each run were discarded in order to allow for equilibration of T1 saturation effects (TR = 2.2 s, TE = 30 ms, sequential acquisition, 38 slices of 2.75 mm, field of view 220 mm, 80 × 80 matrix, in-plane resolution 2.75 mm). After the functional runs a high-resolution 3D T1-FFE scan for anatomical reference was obtained (TR = 9.760 ms; TE = 4.59 ms, flip angle = 8 degrees, 140 slices, 0.875 × 0.875 × 1.2 mm^3^ voxels, field of view = 224 × 168 × 177 mm^3^). Head motion was restricted by using foam inserts between the head and the head coil. Visual stimuli were projected onto a screen in the magnet bore that could be viewed through a mirror attached to the head coil.

### 2.5 Behavioral data analysis

The use of response selection strategies on the marble task was evaluated by computing the Random Number Generation 2 (RNG2) index using Towse and Neil's (1998) RgCalc program. The RNG2 index is an adaptation of the RNG index (Evans, 1978) optimized for two-choice response sequences, which considers the randomness of the sequence (Neuringer, 1986). Scores can range from 0 (no predictability) to 1 (complete predictability).

### 2.6 fMRI data analysis

Data were preprocessed using SPM8 (Welcome Department of Cognitive Neurology, London). Images were corrected for rigid-body motion. Translational movement did not exceed 1 voxel (<3 mm) and mean movement did not differ between age groups, *F* (1, 42) = .043, *p* = .84, *η*^2^ = .001. Structural and functional volumes were spatially normalized to T1 templates. The normalization algorithm used a 12-parameter affine nonlinear transformation involving cosine basis functions, and then resampled the volumes to 3-mm cubic voxels. Templates were based on the MNI305 stereotaxic space (Cocosco et al., 1997), an approximation of Talairach space (Talairach and Tournoux, 1988). Functional volumes were spatially smoothed with an 8-mm full-width-at-half-maximum isotropic Gaussian kernel. Statistical analyses were performed on individual participants’ data using the general linear model in SPM8. The fMRI time series data were modeled by a series of events convolved with a canonical hemodynamic response function (HRF) and the temporal derivatives. The onset of marble motion of each trial was modeled as an event of interest. Separate regressors were defined for white nogo (intentional inhibibition), white go (intentional action), green go (externally guided action), and green omissions (omission on the green marble trials). The trial functions were used as covariates in a general linear model, along with a basic set of cosine functions to high-pass filter (120 Hz) the data. The least-squares parameter estimates of the height of the best-fitting canonical HRF for the different conditions were used in pairwise contrasts. All reported effects consisted of at least 10 contiguous voxels that exceeded a false-discovery-rate (FDR) corrected threshold of *p* < .05, unless otherwise specified.

Region of interest (ROI) analyses were performed to further characterize the involvement of brain regions in the development of intentional inhibition. ROI analyses were performed with the MarsBaR toolbox in SPM8 (Brett et al., 2002) (http://marsbar.sourceforge.net). ROIs that spanned several anatomical regions were masked with marsbar-aal ROIs. For rSTN, ROI analyses were performed on an anatomical rSTN template derived from a study using ultrahigh 7 Tesla scanning (Forstmann et al., 2010). For dFMC, ROI analyses were performed on a 6 mm radius sphere centered on −7, 42, 21 (Kühn et al., 2009).

## 3 Results

### 3.1 Behavior

**Fig. 2:**
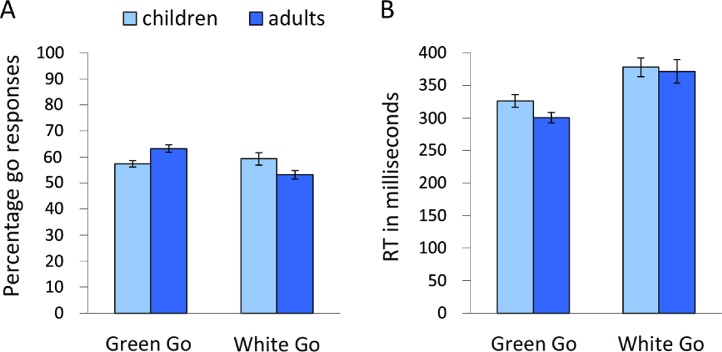
(A) Percentage of go responses in the green and white marble conditions for children and adults separately. (B) Reaction times in the green and white marble conditions for children and adults separately.

The first comparison looked at performance on the instructed green marble trials. The staircase-tracking procedure for green marble trials was overall successful, showing that participants responded in time on 60.63% of the trials (*SD* = 7.06). However, despite the tracking, adults (*M* = 63.22, *SD* = 7.18) were more successful at responding to the green marble in time compared to children (*M* = 57, 35, *SD* = 5.49), *F* (1,42) = 8.68, *p* < .01, *η*^2^ = .17 (see Fig. 2A). Second, we examined the choice behavior on the intentional white marble trials. As instructed, participants decided to inhibit responding to the white marble on approximately 50% of the trials (children: *M* = 40.73, *SD* = 10.03; adults: *M* = 46.83, *SD* = 8.00). However, as can be seen in Fig. 2A children more often responded to the white marble compared to adults, *F* (1,42) = 4.92, *p*< .05, *η*^2^ = .11.

To examine the use of response selection strategies the RNG2 index was computed. A mean RNG2 index of .81 (*SD* = .01) was observed. To examine the randomness of the participants’ choice behavior, the participants’ RNG2 index was compared with a RNG2 index computed over a set of randomly generated sequences of go- and nogo-responses. For the randomly generated set of go- and nogo-response sequences a mean RNG2 index of .801 (*SD* = .002) was observed. Although the RNG2 index for the randomly generated sequences was marginally but significantly smaller compared to the participants’ RNG2 index, *F* (1, 66) = 18.90, *p* < .001, *η*^2^ = .22, these results indicate that the participants’ behavior was close to being random and not driven by simple alternation strategies. Children and adults did not differ in RNG2 index, *F* (1,42) = 1.20, *p* = .28, *η*^2^ = .03, indicating that children and adults did not differ in use of strategies for deciding between responding and inhibiting.

Next, we examined whether there were differences in response times to the instructed green and intentional white marble trials. As expected, reaction times on the green trials were shorter compared to the white trials, *F* (1,41) = 60.79, *p* < .001, *η*^2^ = .60 (see Fig. 2B), indicating that the decision process on the white trials took more time. No main, *F* (1, 41) = .84, *p* = .37, *η*^2^ = .02, or interaction, *F* (1,41) = 1.47, *p* = .23, *η*^2^ = .04, effects of age group were observed.

**Fig. 3:**
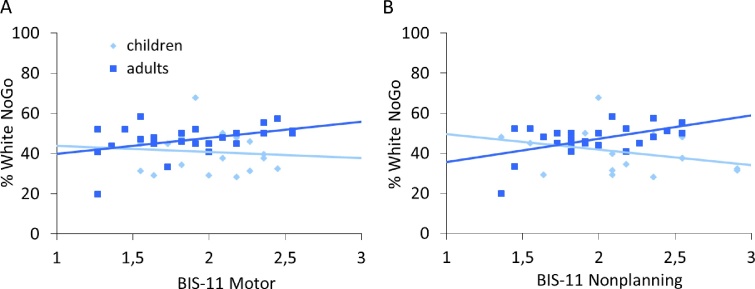
(A) Correlation between intentional inhibition and self-reported motor impulsivity for children and adults separately. (B) Correlation between intentional inhibition and self-reported non-planning impulsivity for children and adults separately.

No developmental differences in self-reported impulsivity on the BIS-11 were observed (all *p*'s > .1) (see Table 1). However, correlation patterns between self-reported impulsivity and performance on the marble task differed between age groups. First, adults who reported more motor impulsivity, more often chose to inhibit, *r* = .409, *p* < .05, but for children this correlation was not significant, *r* = −.084, *p* = .73 (see Fig. 3A). However, a comparison between the correlations showed that the correlations for adults and children did not differ significantly from each other, *z* = 1.06, *p* = .28. Second, adults who reported more non-planning impulsivity, more often chose to inhibit, *r* = .508, *p* < .05, but for children this correlation was not significant, *r* = −.331, *p* = .17 (see Fig. 3B). A comparison between the correlations showed that the correlations for adults and children differed significantly from each other, *z* = 2.72, *p* < .01, showing that the correlation was present in adults but not in children. No correlations between self-reported attentional impulsivity and task performance were observed (all *p*'s > .05).

**Table 1:** Average BIS-11 scores per age group.

	BIS-11 Motor	BIS-11 Non-planning	BIS-11 Attentional
Children	2.01 (.28)	2.14 (.43)	2.19 (.47)
Adults	1.87 (.41)	1.96 (.35)	2.06 (.43)

### 3.2 fMRI

**Fig. 4:**
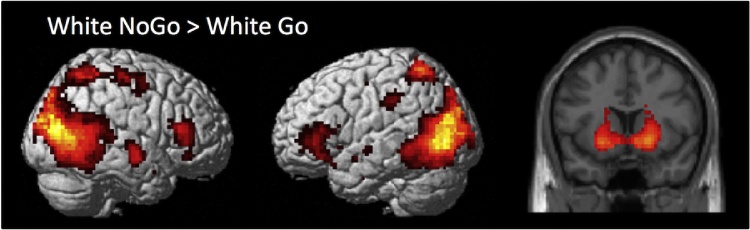
Whole brain contrast showing activation related to intentional inhibition (White NoGo > White Go) for the whole group (*N* = 43) (FDR-corrected *p* < .05, at least 10 contiguous voxels).

To identify the brain regions involved in intentional inhibition, the contrast intentional inhibition > intentional action (White NoGo > White Go) was computed for the whole group (*N* = 43). This analysis revealed activation in a fronto-basal ganglia network, including bilateral IFG, bilateral striatum, and occipital lobe, but not in the STN and the dFMC (see Fig. 4, and Supplementary Table 1 for the MNI coordinates of peak values). A two-sample *t*-test on the whole brain level revealed no developmental differences on the contrast intentional inhibition > intentional action (White NoGo > White Go). To further examine the involvement of the fronto-basal ganglia network in intentional inhibition across development, ROI analyses were performed for the rIFG, right putamen, right caudate, and right globus pallidus.^1^ ROI analyses average across a set of voxels and therefore have more power to detect changes in a priori selected areas.

**Fig. 5:**
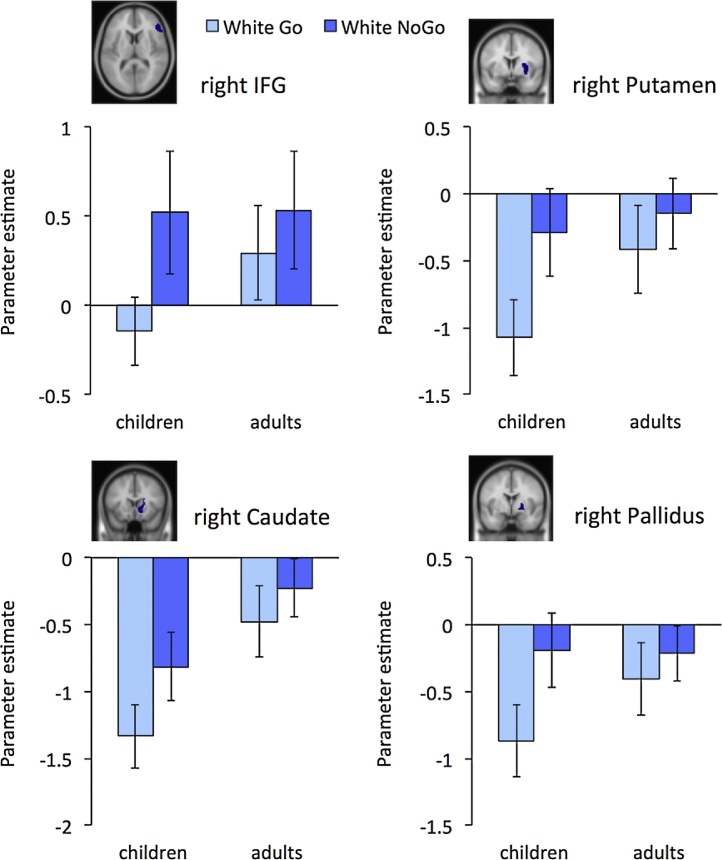
ROI analyses of right IFG, right putamen, right caudate, and right globus pallidus showing activation levels during intentional action (White Go) and intentional inhibition (White NoGo) for children and adults separately.

For rIFG no main effect of age group was observed *F* (1, 41) = .29, *p* = .59, *η*^2^ = .01. However, there was a trend for an age group × condition interaction, *F* (1,41) = 3.71, *p* = .061, *η*^2^ = .08, indicating that children showed significantly more rIFG activation for intentional inhibition compared to intentional action, *F* (1,18) = 25.21, *p* < .001, *η*^2^ = .0 = 58, whereas adults did not, *F* (1,23) = 2.05, *p* = .17, *η*^2^ = .08 (see Fig. 5).

In the right putamen no main effect of age group was observed, *F* (1,41) = .91, *p* = .35, *η*^2^ = .02. However, an age group × condition interaction, *F* (1,41) = 6.92, *p* < .05, *η*^2^ = .14, indicated that the activation difference between intentional inhibition and intentional action in right putamen was larger for children, *F* (1,18) = 29.93, *p* < .001, *η*^2^ = .62, than for adults, *F* (1,23) = 4.03, *p* = .057, *η*^2^ = .15 (see Fig. 5).

For right caudate there was a main effect of age group, showing that children had more stimulus related deactivation compared to adults, *F* (1,41) = 18.09, *p* < .05, *η*^2^ = .10 (see Fig. 5). However, no age group × condition interaction was observed, *F* (1,41) = 2.25, *p* = .14, *η*^2^ = .05.

For right globus pullidus no main effect of age group was observed, *F* (1,41) = .38, *p* = .54, *η*^2^ = .01. However, an age group × condition interaction, *F* (1,41) = 7.28, *p* < .05, *η*^2^ = .15, indicated that children showed relatively more right globus pallidus activation during intentional inhibition compared to intentional action, *F* (1,18) = 23.12, *p* < .001, *η*^2^ = 56, than adults, *F* (1,23) = 2.86, *p* = .10, *η*^2^ = .11 (see Fig. 5).

#### 3.2.1 Role of STN

**Fig. 6:**
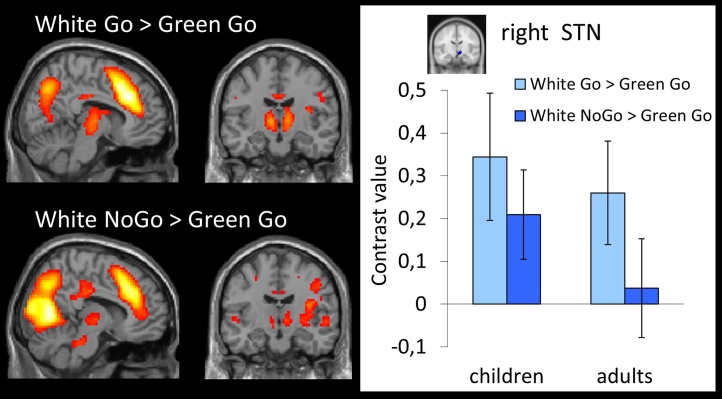
(A) Whole brain contrast showing activation related to intentional action decision process (White Go > Green Go) for the whole group (*N* = 43) (FDR-corrected *p* < .05, at least 10 contiguous voxels). (B) Whole brain contrast showing activation related to intentional inhibition decision process (White NoGo > Green Go) for the whole group (*N* = 43) (FDR-corrected *p* < .05, at least 10 contiguous voxels). (C) ROI analysis of right STN showing activation levels during intentional action decision process (White Go > Green Go) and intentional inhibition decision process (White NoGo > Green Go) for children and adults separately.

No STN activation during intentional inhibition was observed in the contrast intentional inhibition > intentional action (White NoGo > White Go). However, when looking at the intentional inhibition decision process (White NoGo > Green Go) and the intentional action decision process (White Go > Green Go) separately, clear bilateral STN activation was observed for both intentional inhibition and intentional action (see Fig. 6, and Supplementary Table 2 for the MNI coordinates of peak values). To further examine the role of the STN in the intentional inhibition and action processes across development a ROI analysis was performed for right STN. STN activation did not differ between intentional inhibition (White NoGo > Green Go) and intentional action (White Go > Green Go), *F* (1,41) = 2.96, *p* = .09, *η*^2^ = .07. No main, *F* (1,41) = .86, *p* = .36, *η*^2^ = .02, or interaction effects, *F* (1, 41) = .18, *p* = .68, *η*^2^ = .004, of age group were observed (see Fig. 6).

#### 3.2.2 Role of dFMC

No dFMC activation during intentional inhibition was observed in the contrast intentional inhibition > intentional action (White NoGo > White Go). To make sure that there indeed were no differences in dFMC activation, a ROI analysis was performed for dFMC. This analysis showed that dFMC activation did not differ between intentional inhibition (White NoGo) and intentional action (White Go), *F* (1,41) = .43, *p* = .52, *η*^2^ = .01. Also, no main, *F* (1,41) = .12, *p* = .76, *η*^2^ = .003, or interaction effects, *F* (1, 41) = .01, *p* = .94, *η*^2^ = .00, of age group were observed.

#### 3.2.3 Brain-behavior correlations

**Fig. 7:**
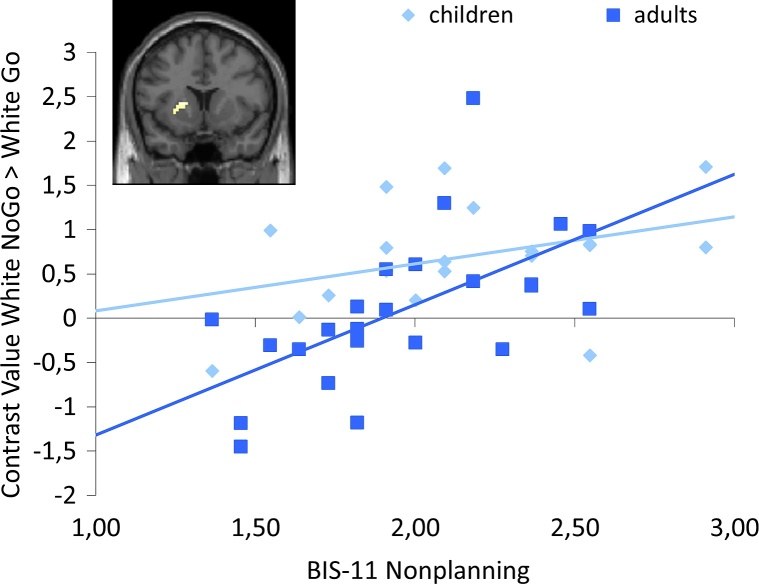
Whole brain regression analysis showing relation between intentional inhibition related neural activation in left putamen and self-reported non-planning impulsivity (uncorrected *p* < .001, at least 10 contiguous voxels). Follow-up ROI analysis showed that this relationship was only significant for adults, not for children.

To examine the relation between self-reported impulsivity and intentional inhibition related brain activation, whole brain regression analyses on the contrast White NoGo > White Go with the BIS-11 subscales as regressors were performed on the whole sample (*N* = 43). No effects were observed at the FDR corrected threshold. However, for non-planning impulsivity a significant relationship with intentional inhibition related brain activation in the left putamen was observed at an uncorrected threshold (*p* < .001, at least 10 contiguous voxels), such that participants who reported more impulsivity showed more activation in left putamen during intentional inhibition (see Fig. 7). A ROI analysis of this left putamen region showed a significant positive correlation in adults, *r* = .600, *p* < .005, but for children this correlation was not significant, *r* = .368, *p* = .12. However, the correlations for adults and children did not differ significantly from each other, *z* = .93, *p* = .35. No relationships between motor and attentional impulsivity and intentional inhibition related brain activation were found.

## 4 Discussion

The present study examined the neural correlates of an important component of self-control; namely intentional inhibition, in children and adults. We used the marble task previously developed by Kühn et al. (2009) to test the neural correlates associated with responding to a rolling marble versus withholding responding to a rolling marble, where the latter process requires intentional inhibition of a prepotent response. As predicted, children and adults showed different recruitment of the fronto-basal ganglia network during intentional inhibition. However, no effects were observed in the dFMC, suggesting that differences are attributable to a similar process as what previously has been observed for externally guided inhibition (see Luna et al., 2010 for a review).

Prior studies have reported that there is a developmental change in inhibitory performance when the task involves external cues (Luna et al., 2010), but much less is known about developmental differences when inhibition is internally driven. The first question we addressed was therefore whether children were able to withhold responding when they had to freely decide to do so. On a behavioral level we show an early development of intentional inhibition performance. That is to say, children intentionally inhibited responding to the rolling marble on approximately 50% of the trials. Also, our RNG2 results indicate that children as well as adults did not use strategies to choose between inhibiting and acting. These findings are in line with our previous studies in which we have shown mature performance levels on intentional inhibition tasks in late childhood/early adolescence on similar tasks (Schel and Crone, 2013, Schel et al., 2013).

Even though both children and adults inhibited responding to the marble on approximately 50% of the trials, it is possible that developmental differences occur in neural responses to intentional inhibition demands, which is potentially a more sensitive index than deciding whether to press the button or not. Therefore, we examined the involvement of the fronto-basal ganglia network in intentional inhibition in children and adults. We show that the fronto-basal ganglia network, which is well known for its involvement in externally driven response inhibition (Aron et al., 2007, Aron and Poldrack, 2006, Jahfari et al., 2011) is activated during both intentional inhibition and intentional action. Although the fronto-basal ganglia network was activated for both intentional inhibition and intentional action, activation in most nodes of the network (i.e. rIFG, right putamen and right globus pallidus) was larger for intentional inhibition. Thus the fronto-basal ganlia network appears to be important for inhibition, irrespective of how inhibition was triggered (externally or internally driven) (see also Schel et al., 2014).

Although there were no developmental differences on the behavioral level, we observed differential recruitment of the fronto-basal ganglia network such that children showed more activation for intentional inhibition compared to intentional action, whereas for adults activation did not differ between intentional inhibition and intentional action. Thus, it appears that children show increased recruitment of the fronto-basal ganglia network during intentional inhibition to reach adult performance levels. The pattern of trend-level increased activation in rIFG and increased activation in putamen during intentional inhibition in children compared to adults is consistent with some studies on externally driven response inhibition (Casey et al., 2002), but not with others which have reported less recruitment of rIFG and instead compensatory recruitment in dorsolateral prefrontal cortex (Bunge et al., 2002, Durston et al., 2002, Luna et al., 2010). In this study, we do not find compensatory recruitment for children outside the fronto-basal ganglia network. Thus, children already activate the same network as adults, but to a different extent. One explanation for this pattern of results might be that for adults acting and inhibiting are two equal response options, whereas for children acting is the default response. Therefore, overruling this default response in order to intentionally inhibit, might require increased recruitment of the fronto-basal ganglia network.

One notable finding was that the STN was not more active for intentional inhibition, even though it has been consistently reported as an important region for externally driven inhibition. However, additional analyses showed that the STN, which is conceptualized as a main inhibitory node in the fronto-basal ganglia network for inhibition, was equally activated for intentional inhibition and intentional action. These findings corroborate the conceptualization of response inhibition and response selection as “two sides of the same coin” of Mostofsky and Simmonds (2008). Inhibition and action can be seen as two equal response options, which are supported by a similar neural network (Karch et al., 2009, Mostofsky and Simmonds, 2008).

Finally, we show that individual differences in self-reported impulsivity are predictive of intentional inhibition performance and the underlying neural correlates. Interestingly, the results of our exploratory analyses into the relation between self-reported impulsivity and intentional inhibition show that people who are more impulsive more often chose to inhibit. Also, they showed more activation in the left putamen during intentional inhibition (note that these results are based on an uncorrected threshold of .001, >10 contiguous voxels). This is opposite to the relation observed between impulsivity and externally driven response inhibition; there more impulsivity is predictive of worse inhibitory performance (Casey et al., 2011). The positive relation between self-reported impulsivity and intentional inhibition could suggest that impulsivity is not always a negative trait. When provided with a choice, impulsive people are well able to choose to inhibit. Thus, acting does not always appear to be the default choice. However, it should be noted that in the current study, the choice between inhibition and action was made in a relatively neutral context (i.e. there were no consequences depending on either choice). In daily life, this is often not the case. There the choice between inhibition and action is almost always influenced by motivational factors like loss, reward or punishment (Leotti and Wager, 2010). How impulsivity influences intentional inhibition in those circumstances remains an important question for future research.

A continuing debate in research on intentional inhibition is the role of the dFMC. Some studies, including the study by Kühn et al. (2009), which also used the marble paradigm, have shown that the late veto of a response is associated with more activity in the dFMC. This has been interpreted as a role of this area in the voluntary control of an action (i.e., the “whether” decision in action control) (Brass and Haggard, 2008, Haggard, 2008). Several other studies have confirmed that this region is more active in other paradigms on intentional inhibition, such as choosing not to gamble (Campbell-Meiklejohn et al., 2008) or inhibition of cigarette cravings (Brody et al., 2007). However, not all studies can replicate this effect, for example, in a prior study in adults we observed dFMC activation only under specific preceding context conditions (Schel et al., 2014). In the current study, we found no evidence for dFMC activation in children, suggesting that neural differences are only observed in the network which is also engaged by externally triggered inhibition.

Some limitations of the present study deserve mentioning. First, we only included a selected age-range (10–12 year-olds and 18–26 year-olds) in the present study. Future studies should include participants across the whole age range from early childhood to adulthood, to examine whether there are specific developmental periods during which intentional inhibition is most sensitive to developmental change (see also: Schel and Crone, 2013). Second, we cannot completely rule out the possibility that participants might have pre-decided not to initiate an action, instead of canceling a prepared action at the last moment on the intentional inhibition trials. However, the fast paced and unpredictable nature of the task, made it very difficult to pre-decide. In future research electromyography measures might be included, to make sure that initial action preparation is also present on intentional inhibition trials. Third, since the present study did not have an externally guided inhibition condition, we could not directly compare the development of intentional versus externally driven inhibition. Follow-up studies should include an externally driven inhibition condition to be able to directly compare intentional and externally driven inhibition. Fourth, the present study used the relatively neutral marble task, in which there are no strong internal motivations for action. An interesting avenue for future research might be to include a strong affective or motivational component, such as is the case in the marshmallow paradigm (Mischel et al., 1989). This might be especially interesting for research in adolescence, when there is a sensitivity towards rewards (Somerville et al., 2011). Fifth, although the role of the rIFG is typically interpreted in terms of response inhibition (for a review, see Ridderinkhof et al., 2011), alternative conceptualizations in terms of action selection have also been proposed (Verbruggen et al., 2010). While the present study does not allow to distinguish the role of rIFG with respect to those alternatives, they may be reconciled by pointing to the role of rIFG in overriding one response alternative (here, to act) in favor of another (here, to inhibit).

To conclude, the present study was the first to examine the neural correlates of intentional inhibition in children and adults. Although children performed at adult level, children showed increased recruitment of the fronto-basal ganglia network during intentional inhibition. Individual differences in impulsivity were predictive of intentional inhibition, more impulsive people were more likely to choose to inhibit. This research has implications for research into disorders of impulsivity, such as ADHD, which are currently associated with poor self-control abilities (Scheres et al., 2006, Scheres et al., 2010). Many types of impulsive behaviors, which are typical for children and adults with disorders of impulsivity, require an intentional decision to inhibit, given that external cues are not always available.

## Conflict of interest

There is no conflict of interest.

## Footnotes

1 Here we only report right hemisphere ROIs in line with previous response inhibition literature. However, for the left hemisphere ROIs similar results were found.
