# Supplementary material for: Choosing not to act: Neural bases of the development of intentional inhibition
Source: Dev Cogn Neurosci. 2014 Aug 20;10:93–103. doi: 10.1016/j.dcn.2014.08.006 (PMC6987865; doi:10.1016/j.dcn.2014.08.006)
Supplement: Supplementary file 1 [file mmc1.doc]

**Supplementary Table 1**

Brain regions revealed by the whole brain contrast intentional inhibition > intentional action (all FDR corrected, *p* < .05, > 10 voxels) for the whole group (*N* = 43).

| Anatomical region | L/R | K | Z | MNI coordinates | | |
| --- | --- | --- | --- | --- | --- | --- |
|  |  |  |  | x | y | z |
| **Intentional inhibition > Intentional action (White NoGo > White Go)** | | | | | | |
| Occipital lobe | L/R | 11247 | 7.12 | 24 | -78 | 21 |
| Putamen | R |  | 5.17 | 18 | 6 | -9 |
| Putamen | L |  | 4.69 | -21 | 12 | -9 |
| Superior Temporal Gyrus | R | 104 | 4.86 | 60 | -9 | -6 |
| Inferior Frontal Gyrus | R | 148 | 3.98 | 54 | 33 | 9 |
| Supramarginal Gyrus | L | 47 | 3.51 | -63 | -24 | 36 |
| Superior Temporal Gyrus | L | 25 | 3.08 | -45 | -9 | -6 |
| Precentral Gyrus | L | 14 | 2.96 | -30 | -12 | 48 |

**Supplementary Table 2**

Brain regions revealed by whole brain contrast, focused on decision processes (all FDR corrected, *p* < .05, > 10 voxels) for the whole group (*N* = 43).

| Anatomical region | L/R | K | Z | MNI coordinates | | |
| --- | --- | --- | --- | --- | --- | --- |
|  |  |  |  | x | y | z |
| **Intentional inhibition > Externally driven action (White NoGo > Green Go)** | | | | | | |
| Occipital Lobe | L/R | 17071 | 7.65 | 12 | -66 | 3 |
| Middle Cingulate Cortex | L | 320 | 4.97 | -6 | -27 | -33 |
| Caudate Nucleus | L | 79 | 4.35 | -6 | 9 | 12 |
| Thalamus (including STN) | R | 77 | 3.56 | 9 | -12 | 0 |
| Thalamus (including STN) | L | 24 | 3.33 | -18 | -30 | 3 |
| Postcentral gyrus | L | 15 | 3.16 | -60 | -6 | 24 |
|  |  |  |  |  |  |  |
| **Intentional action > Externally driven action (White Go > Green Go)** | | | | | | |
| Middle Cingulate Cortex | L/R | 6165 | 7.58 | 6 | 27 | 33 |
| Supramarginal Gyrus | R | 849 | 5.98 | 51 | -42 | 42 |
| Precuneus | R | 946 | 5.48 | 9 | -66 | 42 |
| Inferior Parietal Lobe | L | 427 | 5.32 | -54 | -42 | 51 |
| Thalamus (including STN) | L/R | 446 | 4.90 | -9 | -12 | 3 |
| Middle Temporal Gyrus | R | 119 | 4.26 | 54 | -27 | -3 |
| Cerebellum | L | 22 | 3.58 | -30 | -66 | -27 |
| Insula | R | 14 | 3.39 | 36 | -12 | 15 |
| Superior Temporal Gyrus | L | 36 | 3.20 | -39 | -30 | 12 |
